# Case of Phlebitis Successfully Treated

**Published:** 1841-02

**Authors:** George Bell


*Case of Phlebitis successfully treated.* By George Bell, M. D., &c.—Phlebitis is a disease over which local remedies have little control, and one whose general management does not appear to be accurately understood. Cases of successful treatment, therefore, are alike interesting and important, for it is from their multiplication alone, that correct principles can be deduced. In order to assist in the fulfilment of this object, we record the following history:

Richard Millar, aged 22, of spare habit, sallow complexion, and habitually temperate, was admitted into St. Bartholomew's Hospital on the 8th of June, 1836, laboring under œdematous erysipelas, of the forearm, and simple integumental inflammation of the upper arm on the right side. His expression was anxious; the skin was hot and dry; the pulse frequent and full; the bowels were constipated; and the other symptoms which usually attend fever were observed.

He stated that, about ten days previous to his admission, he had been bled on account of a pain in his side, that on the following day he felt well, and returned to his work. The integument surrounding the wound made by the lancet became slightly inflamed and painful; but he continued at labor, heedless of the increasing pain and inflammation, until inclination and ability to be active had so diminished that he was obliged to confine himself to the house. His symptoms gradually increased in severity, until the evening of the ninth day, when he presented himself at the hospital in the condition already described. He had not been subjected to any treatment.

Twenty-four leeches were applied to the upper arm; the limb was directed to be fomented; and a cathartic of calomel and the compound extract of colocynth was administered.

On the following day the arm was less painful, but there was no abatement in the swelling, althoug the parts were less tense, and no improvement in the general symptoms. He now, for the first time, complained of pain shooting from the site of the wound towards the axilla; his intelligence was less acute, and his tongue was beginning to be incrusted at the the edges. The basilic vein could be felt indurated nearly throughout its whole extent.

On the 20th, he was in the same state; the wound and the parts in the immediate vicinity were puffy, and obscure fluctuation could be felt. A small incision was made in the tumour, and about one drachm of purulent matter was evacuated. On introducing the probe, it was found that the instrument could pass in two directions only, viz. upwards and downwards, in the direction of the course of the vein, which led to the opinion that the matter had been evacuated from a venous abcess. This opinion was strengthened by the fact, that on gently pressing the vein from above dowards, an additional quantity of tinged purulent matter to the extent of two drachms escaped; and the vein, which previovsly felt hard and tense, had now become comparatively soft and pliant.

His pulse was still rapid, but compressible, and his bowels were inactive; the tongue foul; thirst moderate; and the skin dry but not parched.

A purgative enema was directed to be immediately administered; he was ordered to take every eighth hour a pill, consisting of two grains and a half of chalk and mercury powder, and the same amount of Dover's powder, and to use the effervescing draught according to the symptoms.

21st. The constitutional symptoms were improved; the swelling and pain of the arm were abated; but he complained of pain below the short ribs on the left side, and acute pain in the left knee-joint. The pain of the side was relieved by pressure, but that of the knee was much aggravated by the slightest touch. The knee was fomented. In a few days the mouth was slightly affected by the mercury; the limb regained its normal condition, and the patient as regards general symptoms, was convalescent. The vein was contracted to the size of a violin string. The alterative was omitted so soon as the mouth became affected, and some bitter infusion was prescribed.

On the 28th, he was quite convalescent, and discharged, with a recommendation to go to the country.

This case excited a good deal of interest at the time of its occurrence, and it is an important one in several respects. On the evening of admission it differed in no way from the common case of erysipelas with œdema, for he complained of nothing that could lead us to consider this lesion as secondary, either in importance or event. Our inquiries were directed towards the discovery of *phlebitis*, for considering the wound made by the lancet as the centre, starting point, or the immediate cause of the erysipelas, we thought that it might give rise likewise to inflammation of the vein. This suspicion was not confirmed by aught seen or felt, for the redness and swelling of the upper arm effectually prevented any precise information being acquired by local examination. On the day succeeding that of his admission, matters were somewhat changed; there was pain distinctly referred to the course of the vein, the tenseness of the arm was diminished, and the indurated vein could be felt. Added to this, a tendency to typhoid fever was manifest. A question arose as to the propriety of continuing the antiphlogistic treatment. All the benefit to be expected from depletion or other depressing means had been already conferred, and we doubted the power of such measures, either to arrest or modify the progress of the local disease. Existing signs warranted this opinion, for the violence of the disease was abated, and the force of the constitution was declining; and the event of the following day confirmed it, for purulent matter had been already secreted, and was approaching the surface. We had to do, therefore, with the constitution; we had to deal with the fever which had been awakened.

There is a peculiarity in the fever which attends this and some other local inflammations which cannot be accounted for, either on the tissue affected, or by the intensity of the inflammation. It differs from the fever which accompanies pneumonia, pleurisy, and other simple acute inflammations, which is purely symptomatic, and it is identical with that described by the late M. Broussais, as symptomatic of *gastro enteritis*, or inflammation of the glandular and follicular tissue of the bowels. It has the character of what, in this country, is denominated continued fever; it is little tolerant of depletive measures, and but slightly affected by treatment of the local affection. It is essentially an idiopathic fever, excited by a particular cause, of which cause it immediately becomes independent. We hazard no theory regarding the primary cause of fever, but, having referred to the doctrine of Broussais, shall merely express a doubt whether the fever he recognizes as symptomac of intestinal inflammation, is not rather dependent on disease having existed, by which the general irritability of the system has been so far modified as to render it obnoxious to common causes. In a word, we think that the fever is purely iodiopathic in its nature; that it occurs, not because the gastro-intestinal mucous membrane is depraved, but because it has been depraved; and that the erysipelas, phlebitis, &c. are merely exciting causes, acting on a system predisposed to fever, and waiting an opportunity to exhibit its predisposition.

Having hinted at what we consider to be the nature and origin of fever, it may be expected that something should be said about the nature of the causes of which it is so commonly regarded as symptomatic. A man receives a wound, lacerated, contused, or simple; erysipelas occurs, a degree of fever accompanies it; he is bled, and phlebitis ensues. Little benefit is derived from the depletion, the fever speedily loses any signs of vigor it may have at first exhibited; and it assumes a typhoid type altogether identical with continued fever. This is a case which we have repeatedly witnessed. Here the man was predisposed to erysipelas; an acquired condition of the economy rendered him obnoxious to causes which, to the healthy, are sources of inconvenience rather than of danger. Anterior derangement was proved, by the changed irritability of the part in which the local disease appeared, and the constitution responded by a fever disproportioned in intensity to the exciting cause. Erysipelas is a local disease, recognizing a constitutional origin, and the fever excited by it is idiopathic in essence, originates from the same cause, and is born to immediate independence.

Reversing the order of events, local inflammation frequently arises during the progress of continued fever, and although the fever is its exciting cause, it is primarily dependent on the same anterior vicious working in the system which gave origin to the fever. The occurrence of inflammation in one tissue or organ rather than in another, can only be explained by the doctrine of peculiar susceptibilities. Inflammations occurring in this way are denominated spontaneous, by which term we understand that they arise independently of any influences external to the animal body. If this definition be correct, it implies some anterior change in the diseased capillaries, else when the same stimulus is circulating through the system, why does not general instead of local inflammation arise? It may or may not be true, that fever is another name for general inflammation; but the question still returns, why in addition have we another and a distinct inflammation? One year a large proportion cf the inhabitants of this town were affected with bilious diarrhœa; another year few escaped the depression of influenza; during a third year many above the average suffered the pains of rheumatism; and every year hundreds are seized with continued fever, to which is superadded a local inflammation, termed a complication, and occurring like an epidemic. Some peculiarity in the causes of the epidemic seems to determine the nature of the complication. The constitution has previously been so modified as to render one organ rather than another susceptible of generally acting causes. What may be affected through such a diluted imperceptible agent as these may be produced by the stronger and more sensible operation of unnatural, vicious, or civilized regimen, moral as well as physical. This seems to be the remote cause at once of the local disease and the fever it awakens, and as both are constitutional in origin, our remedies should be chiefly applied through the medium of the constitution. Experience shows that topical remedies have little effect on the local evidences of constitutional ailment, and still less on the fever which the local disease kindles.

Erysipelas may be the exciting cause of phlebitis. A middle-aged man, of dissipated habits, received a wound on the hand; erysipelas ensued, which gradually extended up the arm to the body, so as to involve the shoulder and side of the chest. He died. On dissection it was found that the subcutaneous cellular tissue of the upper arm was affected, the limb was œdematous, the texture immediately surrounding the vein was infiltrated with purulent matter, the coats of the vein were thickened, the *vasa vasorum* were distinct, and the canal of the vessel was filled with purulent matter. We have reason to believe likewise that phlebitis is sometimes the result of burns. We have more than once made minute examination of the veins of subjects brought to the dissecting-room with marks of recent injury from fire. In these cases it was found that the plexus of veins at the bend of the arm, together with the main trunks, were effected in the manner above described. In addition to this there are other reasons for believing that burns sometimes prove fatal by inducing phlebitis. We have observed that burns by which the vitality of the skin is immediately destroyed, are, *cæteris paribus*, less fatal than those which excite such a degree of inflammation, as despite treatment, to advance to mortification or sloughing of that texture. The general symptoms in such cases are similar to those observed in phlebitis; the fever is seldom of a character which demands decided antiphlogistic measures, but, on the contrary, is of such a nature as to require our being on the watch for the time when stimuli are necessary.

We have seen several cases in which the injury was confined to the upper part of the arm, and in which a fever, speedily assuming a typhoid type, set in, and carried off the patient. In comparing such cases with others, in which the vitality of the skin was destroyed by fire, and the patients recovered, we are inclined to believe that the constitutional symptoms were not, in the first class of cases, dependent solely on the integumental inflammation, but that some other local inflammation had arisen which determined the fatal event. The extent of the injury in the case referred to, would not account for so serious a result. One fact, mentioned in the history of Millar's case, enables us to ask whether the practice of re-opening the wound in the vein, with the view to discover and evacuate purulent matter, might not with advantage become more general. We cannot see that it involves risk; and it is reasonable to expect that it might frequently be productive of positive good.

With regard to the treatment of *phlebitis* in general, the practice pursued in the case of Millar seems to be worthy of imitation. It was adopted in his case, because we had frequently seen it successful in cases of erysipelas in which the constitution was similarly disturbed. Local depletion cautiously employed, alteratives, gentle laxatives, and enemata, succeeded by nutrients and a moderate use of stimulants, are the means best calculated to relieve the constitutional suffering, and conduct our patient safely through his convalescence. We say that local depletion should be cautiously employed, because danger may, and often does arise from acting on the imagination, that if it does no good it will do no harm, and we approve of alteratives, because we believe that the severity of the symptoms chiefly depends on a morbid condition of the intestinal and hepatic secretions, which can be most certainly remedied through their influence. We say this, not only because we have seen their beneficial effects in this case, but because we have witnessed their salutary operation in many cases of continued fever, evidently dependent on intestinal derangement; because we have observed their beneficial effects in cases of foul ulcers, and ill-conditioned wounds in which the general symptoms were similar to, though not so intense as those which accompany *phlebitis;* and because we have found them to be valuable agents in many cases where long continued bad health was clearly referable to a depraved condition of the gastro-intestinal mucous membrane, as indicated by the tongue, skin, eye, temper, and the mental and corporal activity of the person. This would be a fitting place to enlarge on sympathy with a view to explain all this. We might talk at length and much more mysteriously about the sympathy which exists between the stomach and other parts of the system. We might quote the numerous propositions of authors, criticise their logic, and express wonder at their powers of supposition. We might consider their data, modify their reasoning, and draw new, perhaps opposite conclusions; but in so doing we should be aiding and abetting in mystifying what has been already sufficiently obscured. Thus, although a fresh hypothesis might be invented, the words explanatory thereof traced, still no one would be benefitted thereby; for it is absurd to speculate on conceptions of whose genuineness we have no certain test. Too much caution cannot be observed in the application of the inductive method of reasoning to the solution of questions connected with a purely practical science.

In the cases alluded to, we dread the active employment of cathartics, unless the bowels are distended with feculent matter, and a necessity evidently exists for their immediate evacuation. In such instances we should employ purgatives with a view merely to unload the bowels, and not with that perseverance which implies a belief in their power to remedy the lesion which determined the lodgement. We here desire that our opinion be not regarded as opposed to the doctrine so ably propounded by the late Dr. Hamilton, whose name is so closely and justly associated with the principle of employing laxatives without purging. Dr Hamilton refers to cases in which it is evident that the symptoms are owing to abdominal irritation produced by the accumulation of feculent matter; and not to cases where they are dependent on a depraved state of the gastro-intestinal mucous membrane, of which the lodgement of fæces is not the most serious effect, or that to which the physician should primarily and principally direct his attention. We employ purgatives in such cases merely to unload the intestines, to remove accumulated secretions, and not with any intention to remedy the lesion which exists, which is more certainly rectified by the judicious employment of mercurial alteratives.

We have frequently observed the co-existence of erysipelas, phlebitis, and other local morbid states, with abdominal derangement; the latter having been known to exist prior to the former, or the fever which accompanies them, and, therefore, independent of both. The frequency of the coincidence awakens a belief in their connection; and the result of treatment directed towards remedying the abdominal lesion justifies the opinion, that that connection is relationship. We have spoken of alteratives as a class of remedies on whose power to rectify the abdominal derangement we place considerable reliance; and we are the more encouraged to do so, because we have generally observed that slight affection of the gums, or fœtor of the breath, and the commencement of a return towards health, are simultaneous events. Although not quite relevant to the subject, we may be allowed to remark, that, in the treatment of delirium tremens by calomel and opium, abatement of the symptoms has almost uniformly been coincident with the characteristic effects of the mercurial. This has been so much the case, that, to us, it appears a good fact on which to ground our prognosis, however slight the change may be. It is an evidence that the constitution is still susceptible of the influence of remedies.

Derangement of the biliary organ is frequently associated with the lesion of the stomach and bowels referred to. Whether it be affected primarily or secondarily, the result is the same: for once established, the two diseases react on each other, and the discord is kept up. Each lesion becomes a strong reason for the persistence of the other, and our object is to remove one, for thereby we shall more readily silence the other. We do not pretend to know the cause of the primary lesion, for it may or may not depend on the causes to which it is commonly attributed; but of this we are assured, that the diseases may go on, though the primary cause has long ceased to operate, for two lesions exist; they are springs, and the pendulum which vibrates between them, striking alternately agrinst each, is sent back with almost undiminished force. But vital strength will yield to continued calls for its exertion; by degrees this energy will diminish; by degrees his reciprocal action will fade; in time it will entirely cease, and the patient be a miserable dyspeptic. The two lesions are now quite independent of each other, and for their removal a doubtful and experimental and complicated treatment is required.—*Edin. Med. and Surg. Jour.*—*Medical Examiner of* December 16, 1840.

